# How will AI make sense of our messy lives and improve our mental health?

**DOI:** 10.3389/fpsyt.2024.1347358

**Published:** 2024-01-18

**Authors:** Jan Speechley, Michael McTernan

**Affiliations:** DATAMIND - The Hub for Mental Health Informatics Research Development, Swansea, United Kingdom

**Keywords:** artificial intelligence, mental health, patient and public engagement, trust, data

## Introduction

There is a growing belief that Artificial Intelligence (AI) will play a major part in mental health research and the development and delivery of new services. We are told that AI could provide all of us with access to fast, effective, and personalized healthcare. We hear stories about how AI is more effective at diagnosis than healthcare professionals (HCP) (1), yet there is a lack of trust amongst the public in AI. Would you rely on artificial intelligence (AI) to help you with your mental health issues?

Jan and Michael, lived experience experts, share their opinions on how AI could connect, or not, with their mental health issues and sometimes “messy” lives.

## Can you map our messy lives in discrete, tidy data sets?

Our mental health is complicated, you could say it is messy, and the factors that influence it create our life story. Our mental health is ours; it is unique, it is personal and precious to us as individuals. We want healthcare professionals to understand our experiences, how they affect us, as this articulates how our mental health issues have developed and also how they could be improved.

AI is built and trained on the data it receives. More data, from more sources with more detail can result in better outcomes. Could sharing our mental health data really enable the creation of personalized, tailored care for those with mental health issues and help them make sense of their messy lives and the impact on their mental health?

Jan reflects on her “messy life.”

Consider one person's life. Their earliest care can shape their lives, parents with complex mental health issues, a childhood punctuated with parental mental health symptoms and behaviors. Basic things like mealtimes never at the same time or even certain.

Add in life – school – exams - work – relationships – children - friendships – physical health, money and the ‘messy life' takes shape with so many external factors including the weather! My mental health is always better in the Summer and the shorter days and cold weather of Winter exacerbates all my anxiety and depression. Can all of these variables be captured in a way that is useful? More importantly, can you persuade people to make this information available?

The stigma of this, the coping strategies that we employ, the feeling of being weak, of needing to hide how we feel. All this adds to the strain of just living a messy life with all its component parts and its demands on time and energy. The fear of stigma and shame means we are less likely to want to share our messy lives.

The care and treatments we receive can impact our mental health in a negative way and add to our messy life. Jan says, “I was told that my depression was “difficult to treat,” It made me feel it was my fault and “I did not want to get better.” How language is used in the collection of data and the provision of service is vitally important. Labels can be the start of health inequalities and increase stigma. Jan says, “I know now none of it was my fault, but it took me many years and therapies to successfully reach and live with that conclusion.” The term “treatment resistant depression” is a less judgemental and more positive sounding phrase.

Generative AI tools don't “understand” mental health and can deliver inaccurate and misleading answers. So, how can the personalized healthcare services promised by AI would be developed to use the right language for each situation?

Could AI be used to develop new approaches to delivering mental health care that offer alternatives to medication, addressing an individual's messy life, recommending lifestyle changes, and tailored talking therapies? For patients like Jan labeled with “treatment resistant depression” could there be an alternative route to treatment.

## Trust and transparency

Jan and Michael consider the decision to share their data.

If we are considering sharing all our deeply personal, messy life data to improve mental health care services and treatment we have to trust those who use it and this now includes AI and machine learning tools. Right now, most people donot trust AI.

A new study from the BSI describes that while half of us support the use of AI in healthcare to reduce waiting times, there is still a significant lack of trust in AI (2). Almost two-thirds of respondents in the UK believe that “that patients should be informed if an AI tool is being used during the diagnostic process” (2). We think there are a few reasons for this mistrust.

Firstly, the use of AI and Machine Learning (ML) in healthcare seems futuristic, uncertain, and risky for patients. The news is full of individuals telling us how dangerous AI could be for society. So, it is little wonder that we are skeptical about AI being used to provide our health services. Aligned to this there is a lack of understanding by the public about what AI is and how it would be used in healthcare. It's just not been on our radar. The public needs a better understanding of how AI and ML can be used in healthcare, the pros and cons, and the impact that will have on them.

If you believe the hype, AI has the potential to make healthcare more accessible, triaging patients to the right treatments and therapies to meet their needs. AI could provide individuals with a personalized treatment plan based on their symptoms, history and lifestyle, without seeing a healthcare professional.

We value seeing a clinician, we build usually trusting (but not always), relationships with healthcare professionals. Will we build similar trusting relationships with Healthcare AI agents? Can AI replace the relationship that we have with a psychologist or a GP?

We would welcome the advances in diagnosis and treatment that AI and ML could bring. To radically improve mental health care, we would allow access to our healthcare data, but we also need to know that our data is safe and secure. For the public to be comfortable with sharing their data we need to overcome the stigma and personal shame associated with mental health issues.

There are good examples of altruistic giving in healthcare. For example, Michael gives blood, he doesn't know what happens to the blood that he gives but trusts the Blood Transfusion Service (BTS) to use it appropriately. How can people working in mental health research and development gain and maintain our trust?

## Are we motivated to share our mental health data with researchers?

Jan says, “I have so many questions about my options, I would like to help others but is it safe for me and helpful for them?”

The public needs a better understanding of how AI and ML will be used in healthcare. For many people AI is a scary concept and terms like Machine Learning are meaningless. This needs to be articulated and delivered in terms that we understand. Make your messaging about AI and ML accessible and relevant to the public. But donot patronize us, we are experts in the mental health issues that we face and have spent much time and effort understanding our situation and how best to manage it.

Jan asks, “who cares about me and my privacy? Will my data be safe and protected, could it be sold or appear on social media platforms. What rights do I have if it all goes wrong?”

If you want our data then we need to know that it will be used by researchers whose credentials and purpose are checked by gatekeepers, including members of the public. At the same time, we don't want our data locked away and never used. We want to make our data easily accessible to researchers to allow them to make good use of it.

## How will our data be used to help others have better mental health?

Can our data be used to stop others developing poor mental health and the issues we have experienced? Can our data be used to help people learn from our experiences? Could AI unpick our messy lives and create a personalized treatment for me?

Researchers need to make sure that we understand their big vision, tell us that “our data saves lives” and tell us how. Help all of us understand how AI and ML built on our data can change lives by creating a better understanding of the causes of mental health issues, the strategies for prevention and better treatments for issues and symptoms.

## What impact will sharing my data have?

Could real life improvements to care treatment and services in the NHS change our lives? Will there be small gradual changes or a big bang that changes everything? Will AI help or hinder, slow or increase the pace of improvements in mental health care?

Researchers need to help us feel like we are making a difference, give us feedback on how our data has helped, maybe even an annual newsletter. Make it part of the process that researchers accessing our data must report, in a public friendly way, on their research and the impact that it could create. You could go even further and ask people providing access to their healthcare data to vote on priorities, suggest areas for research, or be public contributors or participants in a research study.

## Data bias and inclusivity

How will we guard against bias in the data that is used in research? Underserved and hard to reach communities struggle to access services and their data is often excluded from research. What safeguards can we put in place to make sure that AI creates equitable and accessible data?

## Final thoughts

People with mental health issues deserve, need, and want improved, more personalized health care, treatment and services. They know their own lives and are experts in its detail and content.

There is much work to be done by governments, law, and policy makers and all involved in research using data, AI and machine learning, to encourage us to share our precious personal information, to make us understand they can be trusted, to keep it safe and use it to help us and others in the future.

We need to understand the difference that making our mental health data available will make, we need to feel valued and be shown how it has helped and the improvements that will happen for people with mental health issues.

Change can be frightening and can take a lot of getting used to, we have to see the point of the change and that it will make things better - the use of AI and Machine Learning is rapidly changing our lives. In healthcare this could create better, accessible, personalized services, but we cannot hope to accept them without question. We need to:

- Help the public understand what AI and Machine Learning is.
- Describe to them the potential impact that their data can have on research and treatment and care services development.
- Gain and maintain their trust in how their data will be used.
- Keep the public informed of how their data is helping and the impact it is having.
- Make sure that the public understands how much their input is valued and make them feel part of a process of positive change.

Michael and Jan are members of the DATAMIND Super Advisory Group, they are lived experience experts. Take a look at our work and the public and patient facing resources we have created at DATAMIND (https://datamind.org.uk/), including a glossary of terms used in mental health data science (3) and a data literacy course to help people understand how their healthcare data is used and stored (4).

## Author contributions

JS: Conceptualization, Writing—original draft, Writing—review & editing. MM: Conceptualization, Writing—original draft, Writing—review & editing.
